# Lipid regulation of BK channel function

**DOI:** 10.3389/fphys.2014.00312

**Published:** 2014-08-22

**Authors:** Alex M. Dopico, Anna N. Bukiya

**Affiliations:** Department of Pharmacology, The University of Tennessee Health Science CenterMemphis, TN, USA

**Keywords:** MaxiK channel, protein receptor site, protein-lipid interaction, lipids, electrophysiology

## Abstract

This mini-review focuses on lipid modulation of BK (MaxiK, BK_Ca_) current by a direct interaction between lipid and the BK subunits and/or their immediate lipid environment. Direct lipid-BK protein interactions have been proposed for fatty and epoxyeicosatrienoic acids, phosphoinositides and cholesterol, evidence for such action being less clear for other lipids. BK α (slo1) subunits are sufficient to support current perturbation by fatty and epoxyeicosatrienoic acids, glycerophospholipids and cholesterol, while distinct BK β subunits seem necessary for current modulation by most steroids. Subunit domains or amino acids that participate in lipid action have been identified in a few cases: hslo1 Y318, cerebral artery smooth muscle (cbv1) R334,K335,K336, cbv1 seven cytosolic CRAC domains, slo1 STREX and β1 T169,L172,L173 for docosahexaenoic acid, PIP_2_, cholesterol, sulfatides, and cholane steroids, respectively. Whether these protein motifs directly bind lipids or rather transmit the energy of lipid binding to other areas and trigger protein conformation change remains unresolved. The impact of direct lipid-BK interaction on physiology is briefly discussed.

Large conductance, Ca^2+^/voltage-gated K^+^ (BK, maxiK, slo1) channels result from tetrameric association of α (slo1) subunits (Figure 1). In most tissues, slo1 channels are associated with small accessory proteins termed β subunits. Four types of β subunits have been identified, their expression being tissue-specific (Orio et al., 2002). This mini-review focuses on lipid modulation of BK current observed in cell-free systems and thus, studies supporting direct interactions between lipid and BK proteins and/or their immediate proteo-lipid environment.

**Figure 1:**
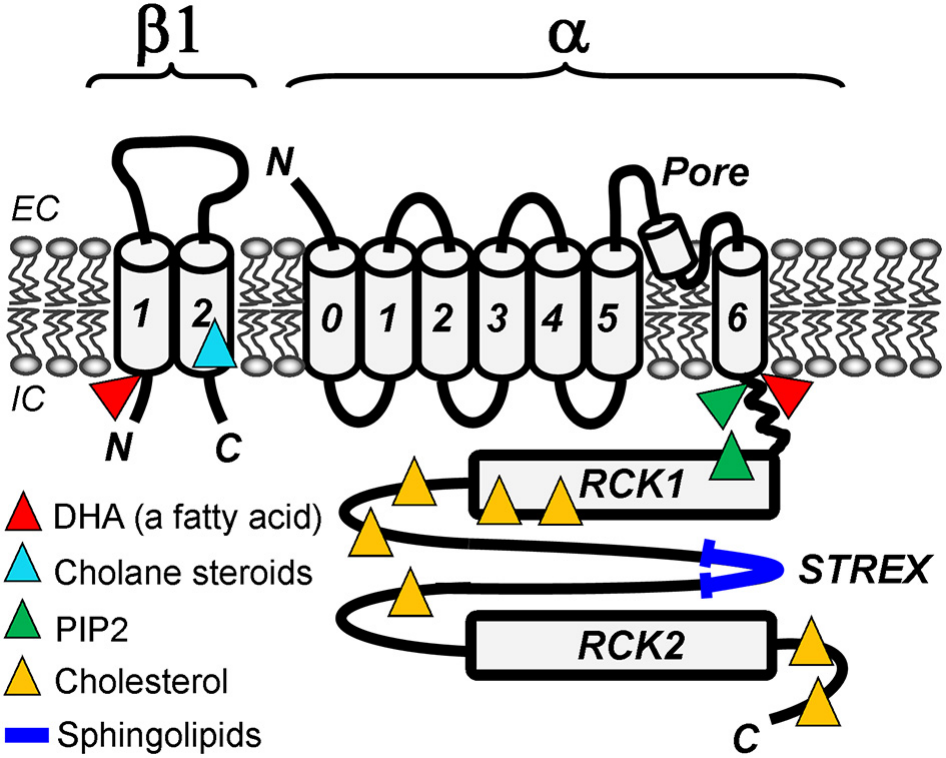
**Lipid-sensing areas in a BK heterodimer made of α (slo1) and β1 subunits.** The cartoon highlights protein regions in which mutations ablate lipid sensitivity of BK channels in cell-free systems, such as excised membrane patches or planar lipid bilayers. Whether these regions directly bind lipids or allosterically modulate BK channel function upon lipid binding to other BK areas remains unresolved.

## Fatty acids

Increase in BK channel activity by low μM FA has been reported in VSM (Kirber et al., 1992; Ahn et al., 1994; Dopico et al., 1994; Clarke et al., 2002, 2003; Martín et al., 2013) and GH3 cells (Denson et al., 2000), and following channel expression in HEK293 cells (Hoshi et al., 2013a-c) and *Xenopus* oocytes (Sun et al., 2007). FA-induced BK activation occurs at a wide range of [Ca^2+^]_i_, and [Mg^2+^]_i_ (Ahn et al., 1994), and in virtual absence of Ca^2+^_i_ (Clarke et al., 2002; Hoshi et al., 2013b). Moreover, FAs neither require voltage-sensor activation (Hoshi et al., 2013b) nor alter the slope of the activity-voltage relationship (Denson et al., 2000). In particular, DHA favors channel dwelling in conducting states by destabilizing the closed conformation of the pore (Hoshi et al., 2013a,b).

FA-induced BK activation does not correlate with changes in membrane fluidity or production of free radicals and oxygen metabolites (Denson et al., 2000). In addition, FA action persists in cell-free, membrane patches (Denson et al., 2000; Clarke et al., 2002; Hoshi et al., 2013b). The membrane-impermeable arachidonoyl-CoA potentiates current only when applied to the cytosolic side of the membrane patch (Denson et al., 2000; Sun et al., 2008), suggesting that the FA-recognition site(s) is accessible from the inner membrane leaflet.

Presence of a negatively charged head-group seems critical for FA “direct” action on BK channels. This action, however, persists after screening membrane surface charge with high-ionic strength solution (Clarke et al., 2002). Structure-activity studies reveal that unsaturated FAs (DHA, arachidonic, and oleic acids) enhance steady-state amplitude and slow inactivation of hslo1+β2 currents whereas saturated FAs fail to do so (Sun et al., 2007). *Cis-unsaturated* FAs increase GH3 cell BK current whereas saturated or *trans*-unsaturated FAs have no effect (Denson et al., 2000). On the other hand, long-chain FAs are more effective than short-chain counterparts in activating VSM BK channels (Ahn et al., 1994; Clarke et al., 2002). The mechanisms and targets underlying differential modulation of BK currents by FA of variant structure remain unidentified.

Channel subunit composition plays a critical role in the final effect of FA on BK current. In the same expression system, DHA potentiates and fails to alter hslo1-mediated and dslo1-mediated current, respectively (Hoshi et al., 2013c). These findings are consistent with the existence of specific, DHA-recognizing sites in slo1 proteins, with hslo1 Y318 playing a critical role in DHA-sensing (Hoshi et al., 2013c). In addition, DHA potentiation of hslo1 current is amplified by BK β1 and β4 subunits (Hoshi et al., 2013a). On the other hand, AA slows inactivation and potentiates current mediated by β2- or β3-containing BK channels. In contrast, long-chain acyl-CoAs facilitate hslo2+β2 inactivation and thus, inhibit overall current (Sun et al., 2008). The presence of opposite charge on residues at positions 11 (N-terminus) and 18 in BK β transmembrane domain-1 (TM1) is crucial for DHA action in presence of β1 and β4 (Hoshi et al., 2013a). It remains unclear whether these residues represent an FA binding site or transduce FA-docking energy into gating modification. Consistent with modulatory or “allosteric” mechanisms, AA inactivates α+β2-mediated currents but fails to affect inactivation of current by the β2-inactivating ball peptide alone, suggesting that AA does not interact with the ball peptide itself (Sun et al., 2007).

The physiological and pathophysiological consequences of BK channel modulation by FAs are under investigation. AA eliminates a transient K^+^ current in neocortical neurons (Sun et al., 2007), which should drastically alter excitability. BK currents mediate AA-induced relaxation of pulmonary artery, yet the exact contribution of a direct FA-BK interaction to this AA action remains unclear (Guerard et al., 2004). However, omega-3 FAs lower blood pressure by directly activating BK channels in VSM (Hoshi et al., 2013b).

## Prostanoids

PGI_2_, PGE_2_, unoprostone and AH13205 activate BK currents in retinal pericytes (Burnette and White, 2006), coronary artery SM (Zhu et al., 2002), HCN-1A (Cuppoletti et al., 2007) and trabecular meshwork cells (Stumpff et al., 2005), respectively. In contrast, U46619 inhibits BK current when the channel is co-expressed with thromboxane A_2_(TxA_2_) receptors in HEK293 cells (Li et al., 2013). PGI_2_- and PGE_2_-induced BK activation require cAMP-stimulated cross-activation of PKG, but not PKA (Hata et al., 2000; Zhu et al., 2002). In VSM, however, PGI_2_ activation of BK current involves a cAMP-independent, Gs protein-dependent component (Tanaka et al., 2004). In turn, U46619 inhibits channel activity in cell-free patches, an action that involves independent associations between channel-forming subunits, BK β1, and TxA_2_ receptors. A direct interaction between prostanoids and BK subunits, however, remains unclear.

Prostanoid-induced BK activation may contribute to the relaxant activity of PGE_2_ in trabecular meshworks (Wang et al., 1998; Stumpff et al., 2005), and PGI_2_-induced VSM relaxation (Tanaka et al., 2004) with consequent retinal vasodilation and blood flow augmentation (Hata et al., 2000). Prostanoid-BK interactions may provide a basis for using PGI_2_-mimetics against pulmonary hypertension (Benyahia et al., 2013). Such interactions may also underlie unoprostone-induced hyperpolarization and consequent protection of cortical neurons against glutamate-induced Ca^2+^_i_ dysregulation (Cuppoletti et al., 2007).

## Epoxyeicosatrienoic acids and leukotrienes

EET and derivatives activate BK channels in VSM (Wu et al., 2000; Zhang et al., 2001; Lauterbach et al., 2002; Archer et al., 2003; Dimitropoulou et al., 2007; Loot et al., 2012) and non-vascular SM (Benoit et al., 2001), cortical collecting duct (Sun et al., 2009), pituitary GH3 (Wu et al., 2000), HEK293 (Fukao et al., 2001), and adrenal chromaffin cells (Twitchell et al., 1997), and crude airway SM microsomes reconstituted into lipid bilayers (Benoit et al., 2001). EET-related epoxyeicosatetraenoic acids (EETe) and 5-oxo-eicosatetraenoic acid potentiate BK current in human pulmonary artery and distal bronchi (Morin et al., 2007, 2009), and cerebral and mesenteric VSM (Hercule et al., 2007). EET and EETe effective concentrations range from nM to low μM (Wu et al., 2000; Benoit et al., 2001; Lauterbach et al., 2002; Hercule et al., 2007; Morin et al., 2009).

EET increases BK current without affecting unitary conductance (Wu et al., 2000; Benoit et al., 2001; Fukao et al., 2001). Rather, EET and 11,12-dihydroxyeicosatrienoic acid (DHET) increase channel open probability (Po) by lengthening open and shortening closed times (Wu et al., 2000; Lu et al., 2001). Modification of gating by EET and EETe is observed across a wide voltage range (Wu et al., 2000; Lauterbach et al., 2002; Hercule et al., 2007) and unaffected by strong buffering of Ca^2+^_i_ (Benoit et al., 2001; Hercule et al., 2007). However, DHET fails to activate BK channels in absence of Ca^2+^_i_ (Lu et al., 2001). EET-induced BK channel activation is suppressed by anti-Gαs antibody (Fukao et al., 2001), and by protein phosphatase 2A inhibitor (Dimitropoulou et al., 2007). However, EET-induced BK activation could be observed in cell-free patches (Wu et al., 2000; Dimitropoulou et al., 2007) and following channel reconstitution into artificial lipid bilayers (Benoit et al., 2001). EET-induced activation of recombinant channels expressed in HEK293 cells does not require β1 subunits (Fukao et al., 2001). Consistently, EETe action on BK channels is preserved in cerebral and mesenteric VSM lacking BK β1 subunits (Hercule et al., 2007). Collectively, these findings point at the BK β1 subunit and its lipid microenvironment as the primary target of EETs and related compounds.

It is noteworthy that 11,12-EET but neither 8,9- nor 14,15-EET, activates BK channels in cortical collecting duct cells (Sun et al., 2009). However, 14,15-EET activates BK channels in inside-out patches from GH3 cells (Wu et al., 2000). In addition, while equipotent in activating coronary artery SM BK channels, several DHETs show a reduced efficacy when compared to 11,12-EET (Lu et al., 2001). Structural specificity in EET action on BK channels is consistent with involvement of distinct EET-recognizing protein sites. In contrast, data from coronary microvessel SM cell-free membrane patches demonstrate a low structural specificity for EET action, as several EET regioisomers and enantiomers, epoxyeicosaquatraenoic, and epoxydocosatetraenoic acids activate BK channels with similar potencies and efficacies (Zhang et al., 2001).

In airway SM, 20-hydroxyeicosatetraenoic acid (20-HETE) and EETs cause membrane hyperpolarization and relaxation of human distal bronchi (Morin et al., 2007, 2009). Likewise, EET-induced BK activation leads to hyperpolarization and dilation of internal mammary (Archer et al., 2003), pulmonary (Morin et al., 2007) and mesenteric arteries (Dimitropoulou et al., 2007). However, EET-mediated SM dilation may be counteracted by EET-stimulated physical association of BK α and β1 subunits in mitochondria: this association enhances mitochondria BK function, leading to loss of mitochondrial membrane potential and thus, depolarization, as reported in pulmonary VSM (Loot et al., 2012). Consistently, EETs fail to hyperpolarize the membrane and relax isolated internal carotid artery (Chataigneau et al., 1998). Finally, BK activation by EET plays an important role in flow-stimulated K^+^ secretion in the cortical collecting duct (Sun et al., 2009), and possibly in regulating adreno-chromaffin cell secretion (Twitchell et al., 1997).

LTA4, LTB4, LTC4, LTD4, and LTE4 (nM-μM) have been tested on β1 subunit-containing recombinant BK channels in *Xenopus* oocyte I/O patches, with only LTB4 significantly increasing channel activity (Bukiya and Dopico, 2013a). This finding raises the hypothesis that BK activation *via* LTB4-BK interaction reduces LT receptor-mediated, SM contraction by LTB4 (Rosenblum, 1985; Lawson et al., 1986; Peters-Golden and Henderson, 2007).

## Cannabinoids

BK channel activation by cannabinoids was detected in myometrial strips (Houlihan et al., 2010), trabecular meshwork cells (Stumpff et al., 2005), ophthalmic artery (Romano and Lograno, 2006), coronary (White et al., 2001) and aortic SM (Sade et al., 2006), and HEK293 cells expressing BK α, α+β1 or α+β4 subunits (Sade et al., 2006; Godlewski et al., 2009). In contrast, μM methanandamide decreases BK activity in mesenteric and aortic SM (Bol et al., 2012). Likewise, virodhamine and synthetic analogs inhibit slo1 channels expressed in HEK293 cells (Godlewski et al., 2009).

The differential effects of cannabinoids on BK activity raised speculation on involvement of several mechanisms and molecular entities in cannabinoid action on BK channels. However, cannabinoid activation of SM BK channels involves neither CB1 or CB2 receptors (White et al., 2001; Romano and Lograno, 2006) nor cannabinoid metabolites (White et al., 2001). Moreover, studies in HEK293 cells rule out involvement of G-proteins and protein kinases (Sade et al., 2006), leading to the hypothesis that a direct cannabinoid-BK channel interaction mediates cannabinoid-induced channel activation (Godlewski et al., 2009). However, methanandamide fails to activate BK channels in cell-free medium (Sade et al., 2006; Godlewski et al., 2009), suggesting that cannabinoid action requires cellular signaling. This signal(s) would likely interact on the slo1 protein, as cannabinoid-induced BK activation is observed in homomeric slo1 (Sade et al., 2006; Godlewski et al., 2009). Interestingly, cannabinoid-induced potentiation of slo1 current is lost after membrane CLR depletion and restored upon CLR repletion (Godlewski et al., 2009), with the slo1 CTD providing several CLR-recognition domains that mediate CLR modulation of slo1 activity (Singh et al., 2012) (see below).

Cannabinoid-induced BK activation seems to play a role in endothelium-dependent vasodilation (White et al., 2001; Romano and Lograno, 2006; Godlewski et al., 2009), modulation of ocular outflow (Stumpff et al., 2005), and myometrial quiescence (Houlihan et al., 2010). In addition, BK activation might contribute to cannabinoid-induced neuroprotection; in particular, to cannabidiol-induced protections against pentylenetetrazol-induced seizure (Shirazi-zand et al., 2013).

## Glycerophospholipids

Glycerophospholipid actions on BK function have been extensively studied in artificial lipid bilayers. Glycerophospholipid-induced changes in unitary conductance (Crowley et al., 2005) and Po (Chang et al., 1995; Crowley et al., 2005; Yuan et al., 2007) have been reported. Increase in slo1 conductance is linked to net negative charge in the glycerophospholipid headgroup (Crowley et al., 2005). In turn, data from bilayers made of variant PCs show that Po decreases with increase in bilayer thickness from PC14:1 to PC 22:1 while increasing from PC22:1 to PC24:1 (Yuan et al., 2007). While this dual profile of Po change is paralleled by changes in mean closed times, BK mean open time increases monotonically with bilayer thickness (Yuan et al., 2007). Moreover, increased open times have been linked to an increase in the glycerophospholipid headgroup cross-sectional area (Chang et al., 1995).

The mechanisms underlying glycerophospholipid-induced modification of BK open and closed times and thus, Po, remain unknown. Putative mechanisms include modification in the physical properties of the lipid microenvironment of the slo1 protein (Chang et al., 1995; Crowley et al., 2005; Yuan et al., 2007); changes in lateral stress imposed by the increasing headgroup size (Chang et al., 1995), perturbation of surface charge density and distribution by negatively charged headgroups (Moczydlowski et al., 1985), and hydrophobic mismatch between protein and bilayer thickness (Yuan et al., 2007). Specific glycerophospholipid-slo1 protein binding cannot be ruled out (Crowley et al., 2005), and gains increasing acceptance as evidence documenting direct binding of membrane lipids to transmembrane proteins keeps growing (Yeagle, 2014).

## Phosphoinositides

PI-induced BK activation has been reported in cerebral artery and skeletal muscle myocytes (Vaithianathan et al., 2008), and with recombinant channels expressed in *Xenopus* oocytes (Vaithianathan et al., 2008; Tang et al., 2014). Phosphatidylinositol 4,5-bisphosphate (PIP_2_)-induced BK activation is independent of PIP_2_ metabolites, and occurs in absence of changes in unitary conductance or voltage-gating. However, this PIP_2_ action requires Ca^2+^_i_. Moreover, PIP_2_ facilitates Ca^2+^_i_-driven gating (Vaithianathan et al., 2008). Very recent work points at the KDRDD loop in the slo1 RCK1 domain as mediator of functional coupling between PIP_2_- and Ca^2+^_i_-regulation of channel activity (Tang et al., 2014): in absence of Ca^2+^_i_, the slo1 RCK1 KDRDD loop decreases the channel's affinity for PIP_2_ whereas in presence of Ca^2+^_i_ the inhibitory modulation of such loop on PIP_2_ affinity is relieved by Ca^2+^-D367 coordination (Tang et al., 2014).

PI-induced BK activation increases with increase in negative charge within the PI headgroup. On the other hand, the more water-soluble analogues diC4 and diC8 are ~10-fold less effective than PIP_2_ in increasing BK activity, a difference that can be explained by their lower affinity to a site(s) and/or by their poor partitioning in the lipid membrane. If membrane partitioning is required for PI to access its site of action, this site should be located in the TM or the intracellular region of the protein, as lipids were more effective when applied to the intracellular side of the membrane. Indeed, the triplet R334,K335,K336 located after S6 in the BK channel-forming cbv1 subunit CTD has been identified as the PI-sensor (Vaithianathan et al., 2008). PIP_2_-induced BK activation is observed in homomeric cbv1 channels and drastically amplified by β1 subunits. Whether this amplification involves PIP_2_-recognition sites in β1 or distinct coupling between β1 and PIP_2_-bound cbv1 is under investigation.

Manipulation of endogenous PIP_2_ levels leads to endothelium-independent, BK-mediated cerebral artery dilation, which suggests that VSM PIP_2_ regulates myogenic tone *via* BK activation (Vaithianathan et al., 2008).

## Lysophospholipids

In I/O patches from an umbilical vein-derived, endothelial cell line, LPI increases BK Po at sub-μM Ca^2+^_i_ and following low basal (pre-LPI) activity while decreasing Po at μM Ca^2+^_i_ and following high basal activity. LPI has no effect in the absence of Ca^2+^_i_ (Bondarenko et al., 2011). The structural bases of LPI-BK interaction and its dependence on Ca^2+^_i_ remain unknown. The gating modifications, however, seem complex, as LPI effect results from changes in both open and closed time distributions. Modulation of BK current by endogenous LPI could play a role in the potentiation of endothelial cell hyperpolarization by low histamine concentrations (Bondarenko et al., 2011).

## Sphingolipids (SPLs)

Sub-μM to low μM SPLs and their metabolites modulate BK activity in pinealocytes (Chik et al., 2001), CHO cells (Chi and Qi, 2006) and an endothelial cell line (Kim et al., 2006). Sulphatides, cerebroside termitomycesphin-A and sphingosine-1-phosphate increase BK current (Chik et al., 2001; Chi and Qi, 2006; Kim et al., 2006; Xu et al., 2011) while ceramides reduce current *via* a PKC-dependent pathway (Chik et al., 2001).

SPL-induced BK current potentiation is dose-dependent, reversible (Kim et al., 2006), and occurs in absence of unitary conductance modification (Xu et al., 2011). SPL action is independent of Ca^2+^_i_ and G protein-coupled receptors (Chi and Qi, 2006; Kim et al., 2006; Xu et al., 2011). Moreover, deletion of the STREX insert in the slo1 CTD reduces channel activation by sulphatides (Chi and Qi, 2006) and totally suppresses the channel's sensitivity to termitomycesphin-A (Xu et al., 2011).

SPL modulation of BK activity could play a role in Ca^2+^ mobilization in endothelial cells (Kim et al., 2006), circadian regulation (Chik et al., 2001), and neuroprotection (Chi and Qi, 2006; Xu et al., 2011).

## Cholesterol, other steroids, and vitamin D

A comprehensive and recent review on modulation of BK channels by CLR and related cholestanes is provided elsewhere (Dopico et al., 2012a,b). In brief, excessive membrane CLR usually decreases BK current, which has been attributed to direct and indirect mechanisms. For decades, CLR action on BK activity has been primarily linked to modification in membrane physical properties by CLR insertion (Chang et al., 1995; Crowley et al., 2003; Lundbaek, 2008). Direct CLR-BK interactions *via* seven CLR-recognition amino acid consensus (CRAC) motifs in the slo1 CTD were proposed (Singh et al., 2012).

In most cases, bile acids and related cholanes, pregnanes, androstanes, and estranes increase BK current, with eventual modification of physiology (reviewed in Dopico et al., 2012a). Later work identified a cholane-recognition site in the BK β1 TM2 where cholane docks *via* hydrogen bonding between its hydroxyl and T169, as well as *via* van der Waals interactions between the steroidal rings and L172,L173 (Bukiya et al., 2011). This site accommodates non-steroidal compounds, such as sodium 3-hydroxyolean-12-en-30-oate (HENA). Cholane and HENA recognition results in endothelium-independent, cerebral artery dilation *via* BK activation (Bukiya et al., 2013b). Because the identified site is found in the SM-abundant β1 and not in other BK βs (2-4), such a site represents an attractive target for rationale design of agents to counteract SM enhanced contraction, as found in asthma, cerebral vasospasm, systemic hypertension, erectile, bladder and uterine dysfunction (Patil et al., 2008; Bukiya et al., 2012).

Considering: 1-the critical roles of both vitamin D and BK channel function in maintaining healthy blood pressure levels (Holtzclaw et al., 2011; Basit, 2013), and 2-the structural similarity of vitamin D with the cholane lithocholic acid, which activates BK channels (see above), it was hypothesized that vitamin D increased BK activity. Indeed, μM vitamin D3 and 25-OH vitamin D3 increase β1-containing, BK-mediated currents after expression in *Xenopus* oocytes (Bukiya et al., unpublished). The consequences of vitamin D action on BK currents are under investigation.

## Conclusions

Modulation of BK current by direct (e.g., independent of cell integrity, signaling or lipid metabolism) interaction between lipid ligand and BK subunits has been reported for a wide variety of lipid species. For some lipids (e.g., cholesterol), lipid-BK channel-forming (slo1) subunit interaction accounts for most of the lipid effect. The majority of lipid-sensing regions in slo1 have been mapped to its intracellular tail domain. Whether these regions directly bind lipids or modulate BK channel function following lipid binding to other slo1 areas remains to be determined. For other lipids (e.g., cholanes), accessory β subunits are necessary for lipid action. Still for others (e.g., PIP_2_), slo1 subunits suffice for lipid action, yet β subunits drastically modify the lipid's final effect. In most cases, the impact of direct BK channel-lipid interaction on organ function is under investigation.

### Conflict of interest statement

The authors declare that the research was conducted in the absence of any commercial or financial relationships that could be construed as a potential conflict of interest.

## Abbreviations

AA: arachidonic acid
BK: calcium- and voltage-gated, large conductance potassium
cAMP: cyclic adenosine monophosphate
Ca^2+^_i_: intracellular calcium
CB: cannabinoid
CHO: Chinese hamster ovary cells
CLR: cholesterol
CRAC: cholesterol recognition amino acid consensus
CTD: cytosolic tail domain
DHA: docosahexaenoic acid
DHET: 11,12-dihydroxyeicosatrienoic acid
EET: epoxyeicosatrienoic acid
EETe: epoxyeicosatetraenoic acid
FA: fatty acid
HEK: human embryonic kidney
GH3: rat pituitary tumor epithelial-like cells
HCN-1A: human cortical neuronal cells
HENA: 3-hydroxyolean-12-en-30-oate
I/O: inside-out patch
LPI: lysophosphatidylinositol
LT: leukotriene
PC: phosphatidylcholine
PG: prostaglandins
PI: phosphoinositide
PIP_2_: phosphatidylinositol 4,5-bisphosphate
PK: protein kinase
Po: open probability
RCK: regulator of conductance for potassium
SM: smooth muscle
SPL: sphingolipid
TM: transmembrane
TxA2: thromboxane A2
VSM: vascular smooth muscle.
